# Development of a ferroptosis-based molecular markers for predicting RFS in prostate cancer patients

**DOI:** 10.1038/s41598-023-50205-1

**Published:** 2023-12-20

**Authors:** Jinquan Chen, Longbin Zhang, Yiling Luo, Chao Tan, Huang Hu, Yuling Jiang, Na Xi, Qinghai Zeng, H. Peng

**Affiliations:** 1 Department of Ophthalmology, The Tongnan District People’s Hospital, Chongqing, China; 2 Department of Ophthalmology, The First Affiliated Hospital of Chongqing Medical University, Chongqing, China

**Keywords:** Cancer, Computational biology and bioinformatics

## Abstract

The goal of this study was to develop a ferroptosis-based molecular signature that can predict recurrence-free survival (RFS) in patients with prostate cancer (PCa). In this study, we obtained ferroptosis-related genes (FRGs) in FerrDb database and clinical transcriptome data in TCGA database and GEO database. Consensus cluster analysis was used to identify three molecular markers of ferroptosis in PCa with differential expression of 40 FRGs, including PD-L1 expression levels. We conducted a new ferroptosis-related signature for PCa RFS using four FRGs identified through univariate and multivariate Cox regression analyses. The signature was validated in the training, testing, and validation cohorts, and it demonstrated remarkable results in the area under the time-dependent receiver operating characteristic (ROC) curve of 0.757, 0.715, and 0.732, respectively. Additionally, we observed that younger patients, those with stage T III and stage T IV, stage N0, cluster 1, and cluster 2 PCa were more accurately predicted by the signature as independent predictors of RFS. DU-145 and RWPE-1 cells were successfully analyzed by qRT-PCR and Western blot for ASNS, GPT2, RRM2, and NFE2L2. In summary, we developed a novel ferroptosis-based signature for RFS in PC, utilizing four FRGs identified through univariate and multivariate Cox regression analyses. This signature was rigorously validated across training, testing, and validation cohorts, demonstrating exceptional performance as evidenced by its ROC curves. Notably, our findings indicate that this signature is particularly effective as an independent predictor of RFS in younger patients or those with stage T III and T IV, stage N0, and in clusters 1 and 2. Finally, we confirmed the expression of these four FRGs in DU-145 and RWPE-1 cell lines.

## Introduction

PCa, the fifth leading cause of death among males globally, is the most widespread non-cutaneous cancer. Radical prostatectomy is the primary curative approach for localized PCa, yet some patients still experience recurrence^1,2^, suggesting potential clinical metastasis and a poor prognosis^1,3^. Early detection of recurrence is critical for determining follow-up treatment strategies for PCa patients. Although some studies investigated various biomarkers or clinical factors predictive of recurrence, no well-established molecular subtypes or prognostic signatures associated with PCa recurrence exist^4^.

Ferroptosis, a form of programmed cell death marked by iron-dependent peroxidation^5^, is especially vulnerable to cancer cells due to their heightened cellular activity^6^. Recently, studies have concentrated on the function of ferroptosis in cancerization and progression^7^. PCa is one of the diseases associated with ferroptosis^8^. Previous research has shown that aberrant lipid homeostasis is related to PCa cancerization and progression to castration-resistant prostate cancer^9^. However, few studies have investigated the correlation between ferroptosis and PCa recurrence or anti-tumor immunology.

In our study, three molecular clusters of PCa, as well as ferroptosis-based signatures predictive for PCa RFS, were identified and robust results validated.

## Results

### Differentially expressed FRGs and functional enrichment

**Figure 1:**
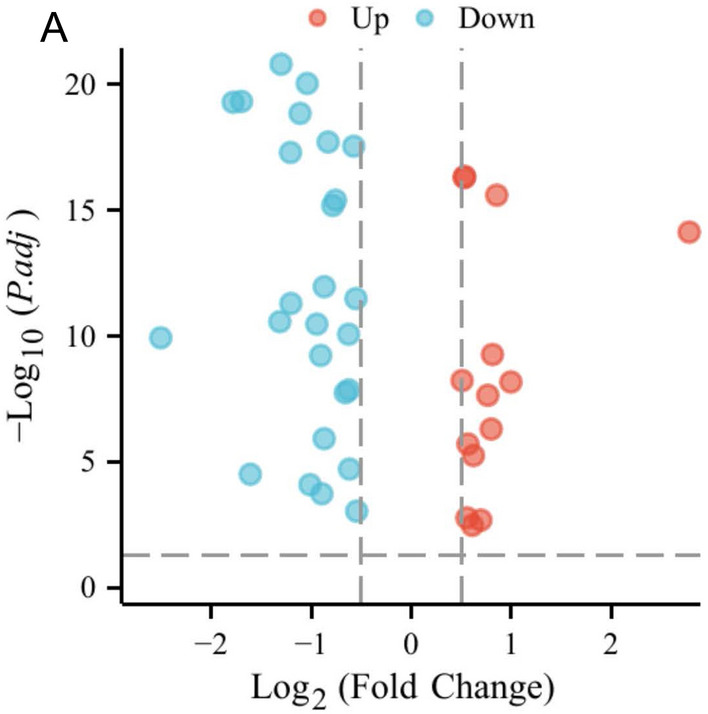
Identification of genes related to ferroptosis that are expressed differently.

**Figure 2:**
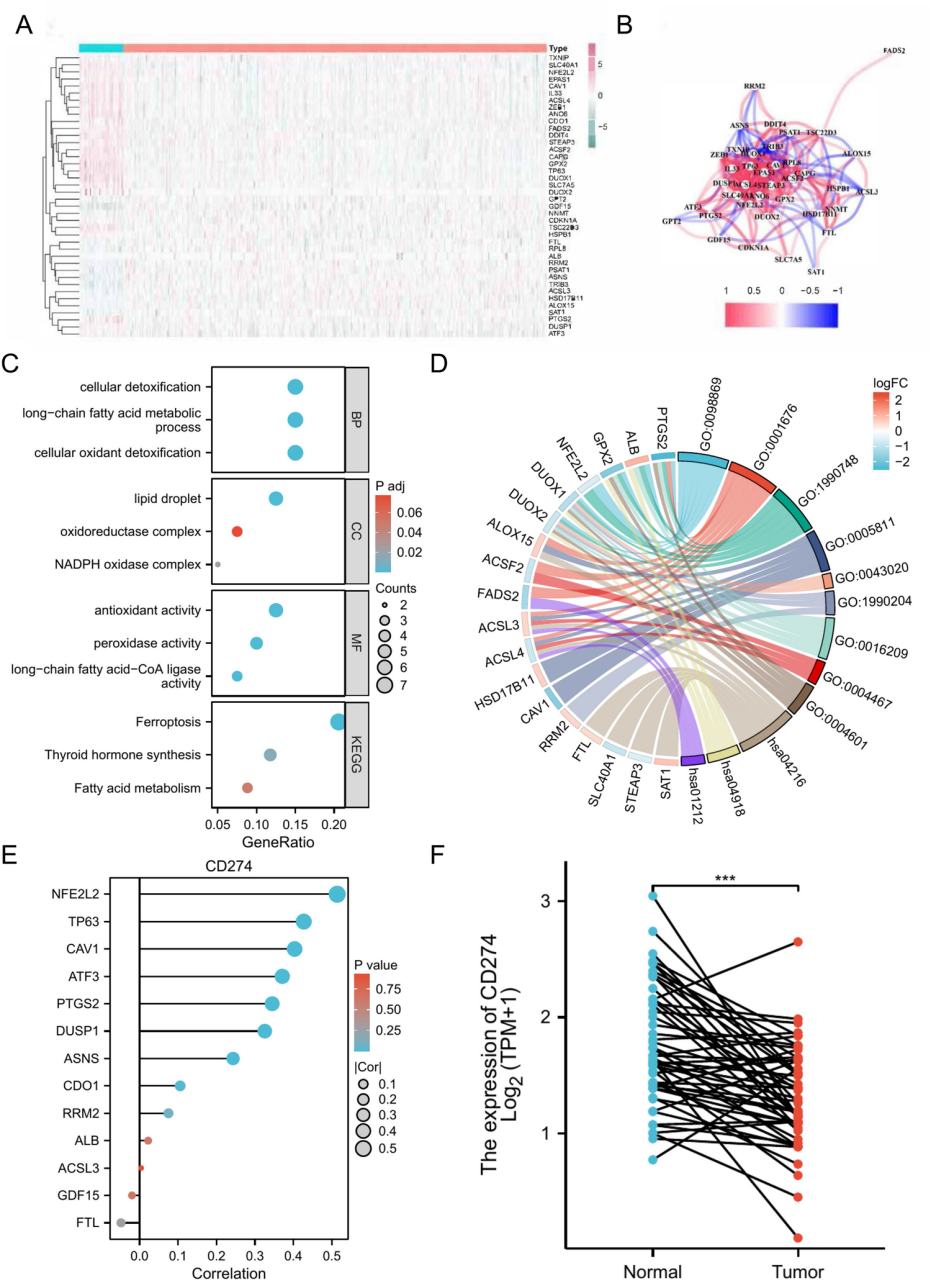
The heatmap (**A**) and correlation network (**B**) of Figure 2.40 illustrate the variously expressed genes associated with ferroptosis, as well as GO (**C**) and KEGG enrichment (**D**). Additionally, (**E**) reveals a correlation between PD-L1 expression and 14 genes related to ferroptosis and of prognostic importance. PD-L1 expression levels in PCa and normal patients (**F**).

To analyze a TCGA cohort of FRGs, the Wilcoxon test was employed to distinguish DEFRGs in TCGA samples that diverged between normal and PCa samples, Fig. 1 shows that a total of 40 functional response genes (FRGs) were identified, of which 26 were down-regulated and 14 were up-regulated. Figure 2A and B display the heat map and correlation network for the FRGs. Functional enrichment analysis of the 40 differentially expressed FRGs (DEFRGs) is shown in Fig. 2C and D. Analysis of the Gene Ontology (GO) showed that oxidative stress and organic acid biosynthesis were the primary biological processes linked to DEFRGs. The cellular component (CC) responsible for these genes was identified as the organelle outer membrane, while molecular function (MF) was peroxides and antioxidant activity. KEGG analysis reveals that these DEFRGs are involved in oxidative stress, organic acid synthesis, fatty acid metabolism, carboxylic acid synthesis, and oxygen response. Univariate Cox regression analysis showed that PTGS2, DUSP1, ALB, ATF3, GDF15, FTL, NFE2L2, RRM2, ACSL3, CAV1, and CDO1 of the TCGA cohort were closely associated with RFS. PD-L1 expression was found to be correlated with the expression of 14 of these FRGs, namely, DUSP1, ATF3, NFE2L2, TP63, CAV1, and ACSL3, as demonstrated in Fig. 2E. In PCa patients, Fig. 2F illustrates a marked decrease in PD-L1 expression in comparison to the normal tissue.

### Three molecular clusters relating to ferroptosis

**Figure 3:**
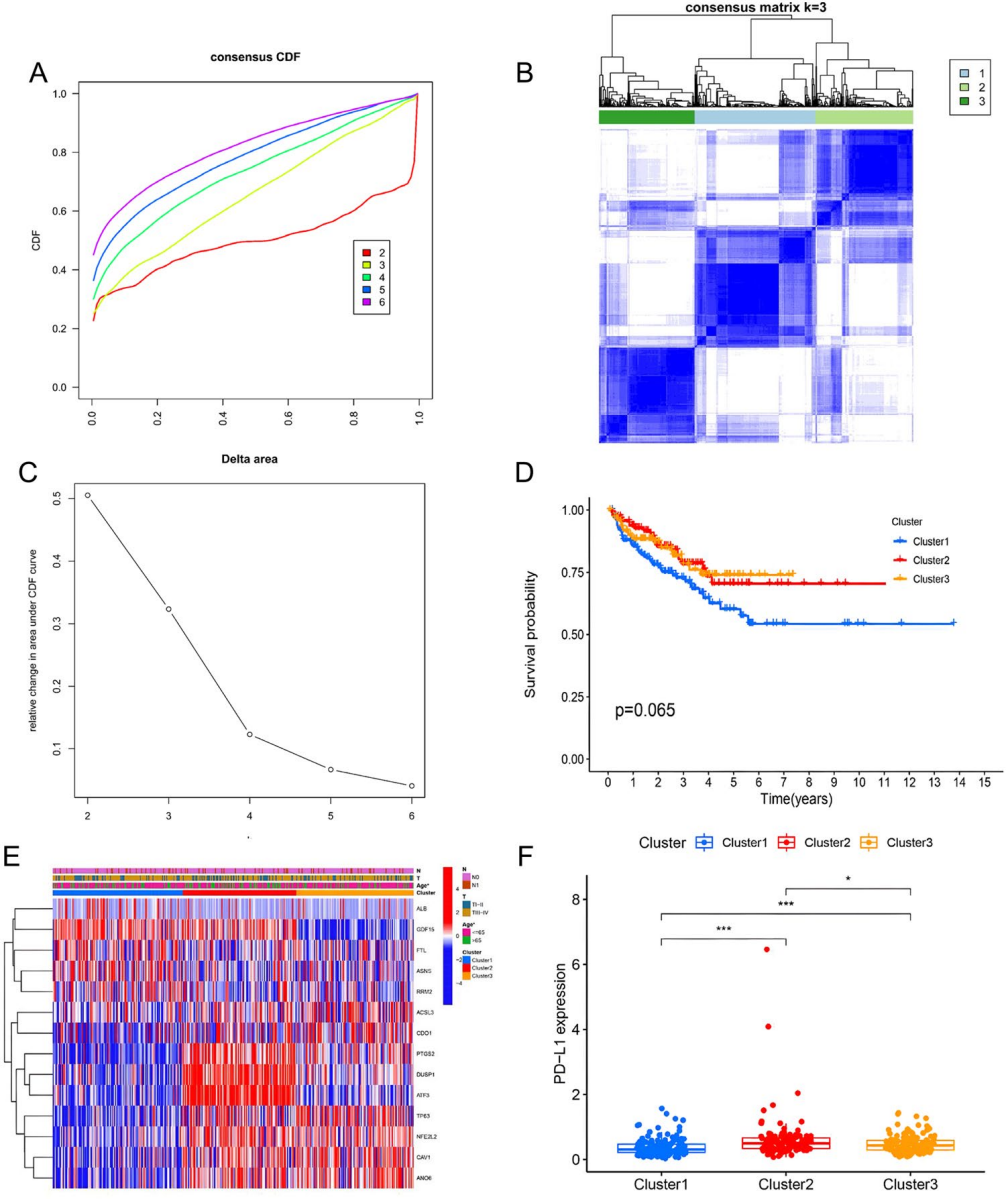
Molecular cluster analysis of molecular clusters of ferroptosis (**A**–**C**). Survival analysis of these three clusters (**D**). Molecular clusters related to biochemical recurrence and clinicopathologic features of free ferroptosis (**E**). PD-L1 expression levels in these three clusters (**F**).

Then, a consensus clustering analysis was used to identify three clusters of ferroptosis. Figure 3A–C demonstrate that cluster 1 had 146 cases, cluster 2 had 121, and cluster 3 had 138. Figure 3D reveals that there were no noteworthy disparities in RFS between these clusters. Figure 3E, however, reveals that the age of the three molecular clusters was significantly disparate. Figure 3F, it is evident that PD-L1 expression was significantly higher in cluster 2 than in clusters 3 and 1 and even more so in cluster 3 than in cluster 1.

### RFS prediction using ferroptosis-based signatures

**Figure 4:**
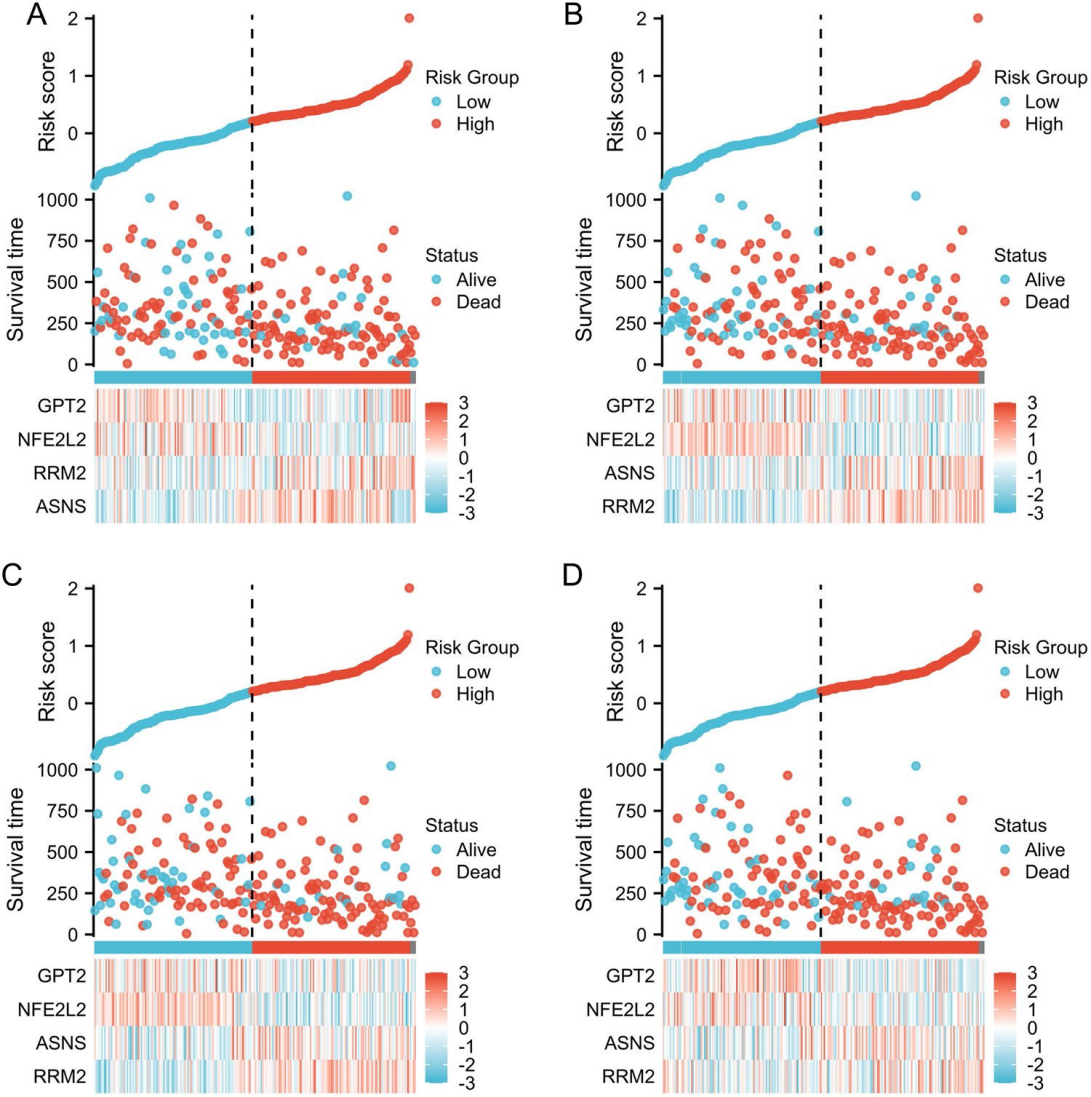
PCa prognostic signature based on ferroptosis. Testing cohort (**A** and **B**) for survival time, (**C**) for the distribution of risk score, and (**D**) for the validation of the TCGA cohort expression heat map, all of which are part of the training cohort (**A**) and (**B**) respectively.

We conducted univariate Cox regression analysis in the training cohort, discovering eight DEFRGs. Subsequently, we employed stepwise multivariate Cox regression analysis to uncover a novel ferroptosis-based prognostic signature, based on the results of the univariate analysis. The final model included only four DEFRGs. The risk score for each case in the training, test, TCGA, and validation cohorts was determined using these formulas; all patients were then divided into high/low-risk groups based on the risk score. Figure 4 shows survival time distributions, risk scores, and expression heat map for training cohorts, testing cohorts, and TCGA cohort.

**Figure 5:**
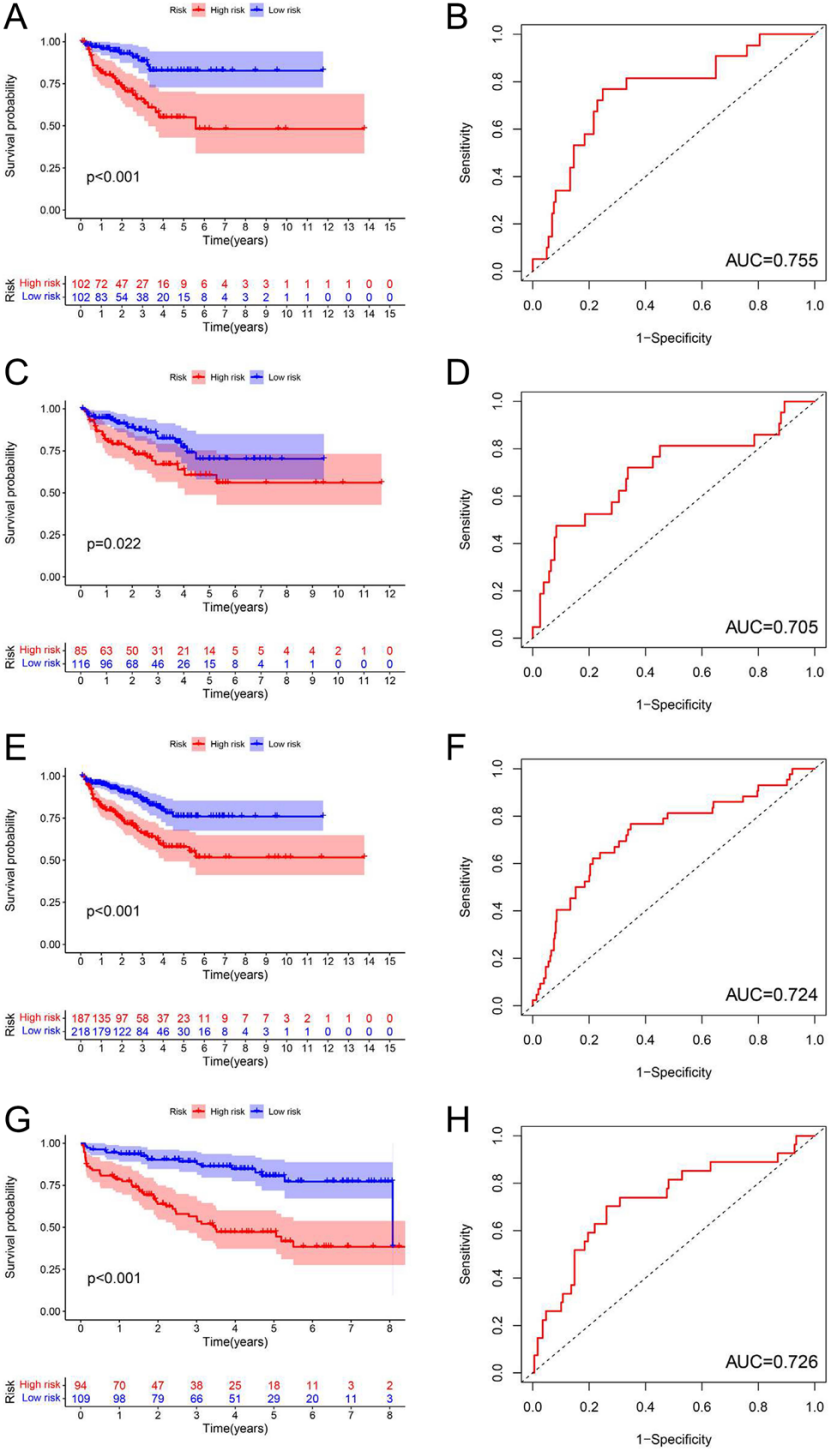
A survival analysis of high and low risk groups in training cohorts (**A**) and (**B**), testing cohorts (**C**) and (**D**), the entire TCGA cohort (**E**) and (**F**), and validating cohorts (**G**) and (**H**) was conducted, and the area beneath the ROC curve was also examined for all of them.

Significant differences in RFS between the low-risk and high-risk subgroups were observed in the training cohort (p < 0.001), testing cohort (p < 0.022), TCGA cohort (p < 0.001), and validating cohort (p < 0.001). Patients with a high-risk score had significantly poorer RFS and a higher likelihood of developing RFS in the training, testing, control, TCGA, and validating cohorts. Based on the outstanding performance of the discovered markers in predicting RFS in PCa, the ROC curve was 0.757,0.715,0.732, and 0.726 for the training cohort, the test cohort, the TCGA cohort, and the validation cohort, respectively (Fig. 5). An independent prognostic analysis revealed that the new signature of ferroptosis could predict BCRFS of PCa univariately and multivariately.

### Subgroup survival analysis

**Figure 6:**
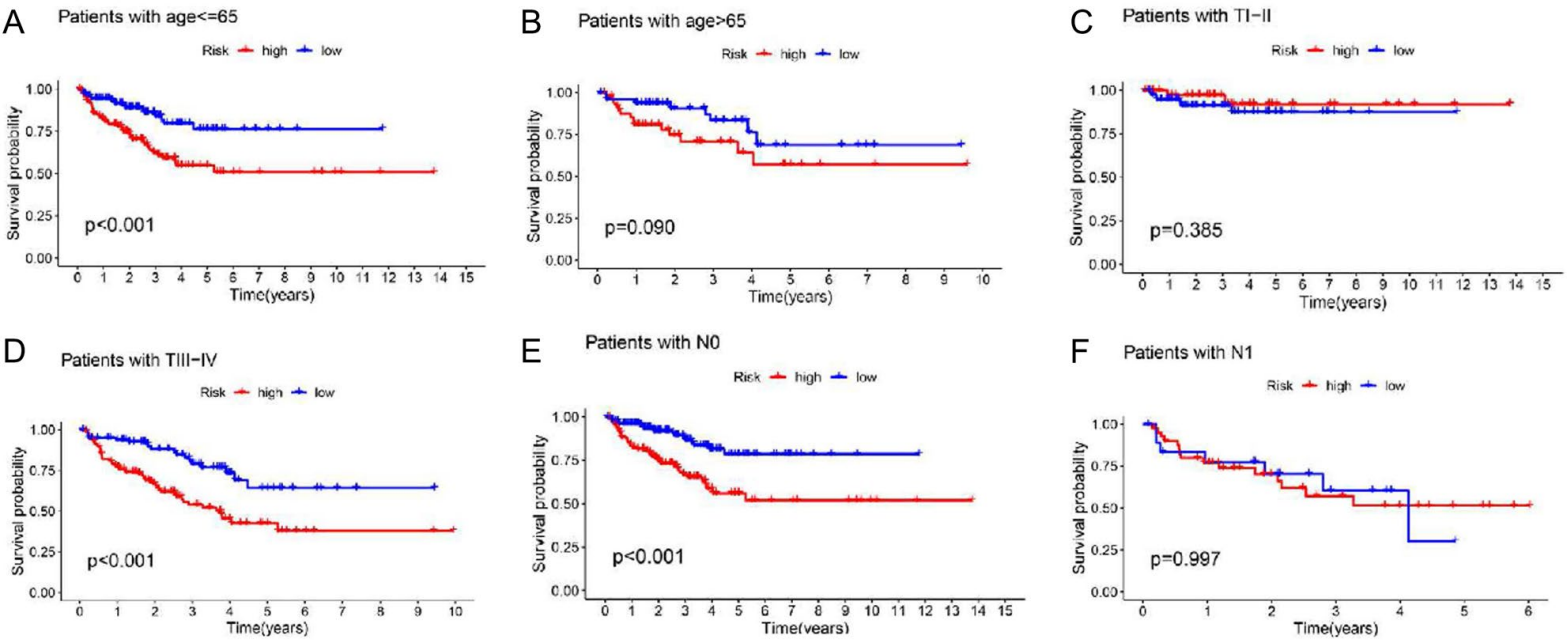
Comparison of subgroup survival in patients aged 65 and over 65 (**A** and **B**), stage T I and stage T II (**C**), stage T III and stage T IV (**D**), stage N0 (**E**), stage N1 (**F**).

An analysis of subgroup survival revealed that the signature of ferroptosis proved especially successful in forecasting RFS in younger patients, those with stage T III and stage T IV, and those with stage N0 PCa. For PCa patients aged 65 or over or in T stage III and T stage IV or N0 stage, low-risk scores were significantly linked to good RFS, while high-risk scores were linked to poor RFS. No statistically significant distinction was observed between low- and high-risk groups of patients aged 65 or above, with stage T I and stage T II or stage N1 (Fig. 6) in regard to RFS.

### Functional enrichment

**Figure 7:**
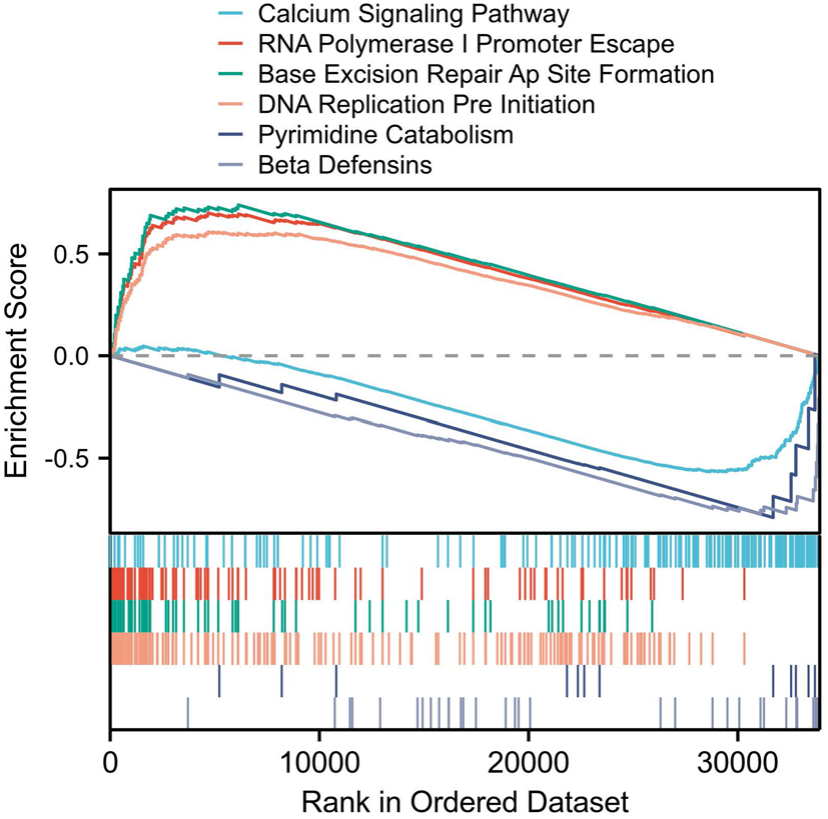
Functional enrichment associated with ferroptosis-based signatures.

The GSEA method revealed a distinction in functional performance between high- and low-risk groups. Those of high-risk had a heightened KEGG pathway for RNA polymerase, DNA replication, and mismatch repair, whereas those of low-risk exhibited heightened aldosterone signaling, calcium signaling, beta alanine metabolism, adipocytokine signaling, and nicotinate/nicotinamide metabolism activity (Fig. 7).

### qRT-PCR and western blotting validation of DEFRG mRNA and protein expression levels in DU-145 PCa cell line.

**Figure 8:**
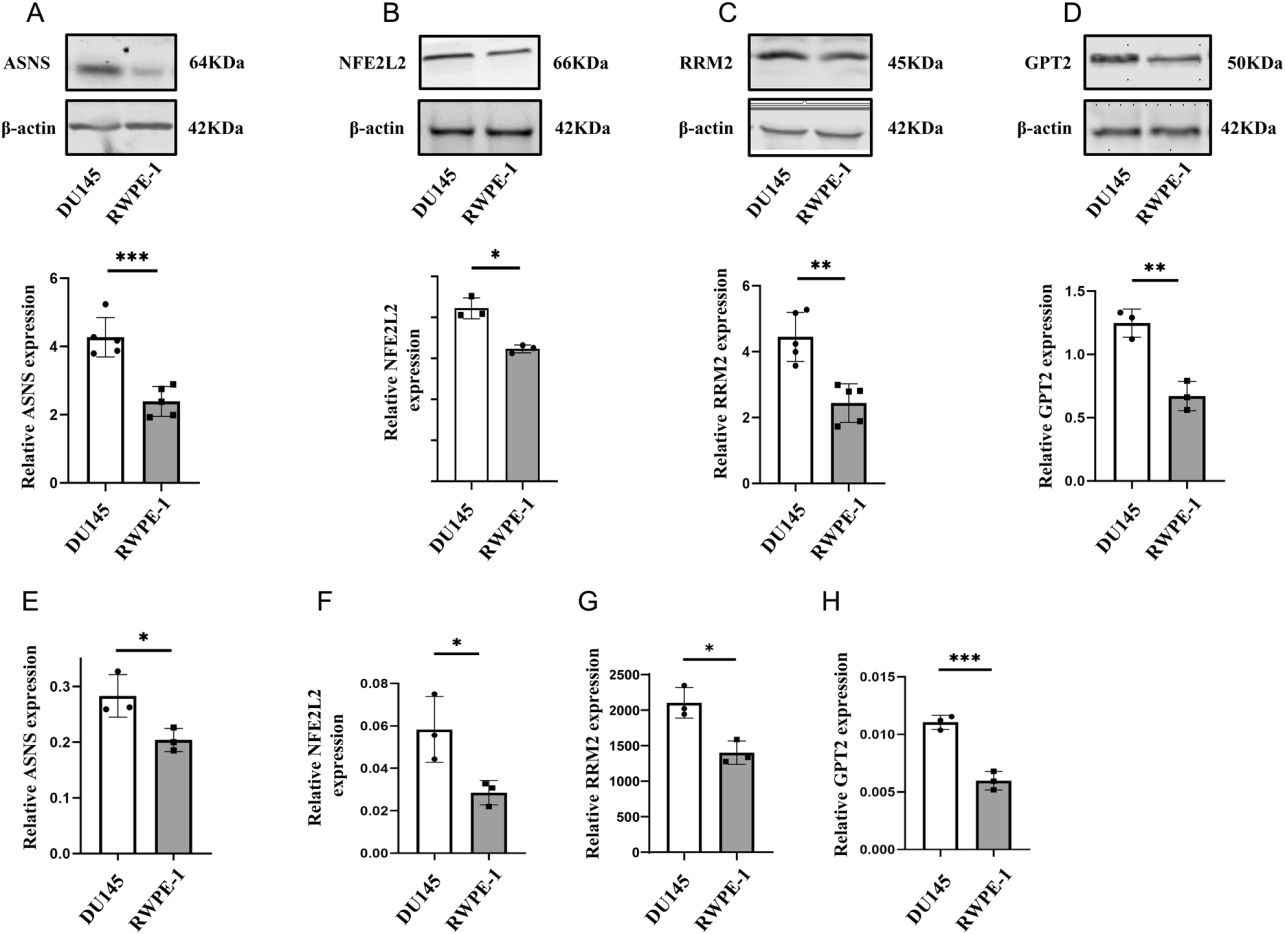
Protein expression levels of ASNS, NFE2L2, RRM2, and GPT2 (**A**–**D**) were compared between PCa cell line and normal prostate epithelial cell line. A comparison of ASNS, NFE2L2, RRM2, and GPT2 (**E**–**H**) in PCa cells to those in normal prostatic epithelial cells was made, with p-values of less than 0.01 and less than 0.001 respectively.

Western Blot (Supplementary Material 1) and qRT-PCR revealed a noteworthy rise in protein expression (Fig. 8A–D) and mRNA expression (Fig. 8E–H) of ASNS, GPT2, RRM2, and NFE2L2 in DU-145 PCa cell line in comparison to RWPE-1 normal PCa cell line.

## Disscusion

PCa is associated with ferroptosis, according to previous studies^8^. It may be possible to induce ferroptosis as a new therapeutic strategy for advanced PCa^8^. PCa's hypersensitivity to ferroptosis is significantly induced by treatment-induced lipid uptake and remodeling^10^. By regulating the polyunsaturated fatty acid oxidation, Nassar et al. Showed that DECR1 protects PCa cells from ferroptosis^11^. After radical treatment of PCa, recurrence is considered a key event^1^. 20–40% of PCa patients experienced recurrence within 10 years; recurrence signified recurrent cancer. Despite curative treatment^12^. It is important to recognize that recurrence on its own does not necessarily impact quality of life or overall survival^13^, recurrence indicates a high probability of metastatic disease^14^. Few studies have explored whether ferroptosis is associated with PCa recurrence.

In this study, we identified three molecular clusters based on ferroptosis, which differed from previous studies. The clusters revealed distinct clinical traits, PD-L1 expression levels, and tumor immune microenvironment features. The objective of our multivariate Cox regression analysis was to predict RFS for patients with PCa based on ferroptosis and four DEFRGs. Verification of signatures having been completed, all patients with high-risk scores from the training, testing, TCGA, and validation cohorts had positive outcomes. The ROC curve was 0.757,0.715,0.732, and 0.726 for the respective cohorts, indicating the signature's suitability for predicting PCa RFS. A multivariate and univariate independent prognostic analysis revealed that ferroptosis-based signatures were independent predictors of RFS for PCa.

The four genes involved in this ferroptosis-based prognostic signature were ASNS, GPT2, NFE2L2, and RRM2. According to Kanishka Sircar et al., it might be possible to target CRPC cells using ASNS inhibitors^15^. Using simultaneous inhibition of GPT2 and OGT, Harri M Itkonen et al. demonstrated that PCa cells could be killed by suppressing growth, viability, and death responses^16^. A study by Qiang Ju et al. found NFE2L2 to be an underlying prognostic marker and related to immunosuppression in low-risk glioma^17^. Zhang et al. suggested that the gene RRM2 may mediate the liver cancer ferroptosis signature^18^. Finally, Mazzu et al. indicated that RRM2 may contribute to immune escape and aggressive PCa^19^. A note of importance is that no information is currently available on the relationship between ASNS, GPT2, NFE2L2, RRM2 and ferroptosis in PCa.

As a result of its retrospective design and limited sample size, this study may suffer from selection bias Since this study used a retrospective approach, prospective real-world evidence is needed for validation of the ferroptosis-based molecular signature presented in this study. The mechanisms underlying ferroptosis, pharmacotherapy, and recurrence need to be investigated further in wet lab studies in vitro and in vivo.

It was found that 40 FRGs were differentially expressed in PCa, as well as three ferroptosis-related molecular clusters. Using four ferroptosis-based molecular signatures (ASNS, GPT2, NFE2L2, RRM2), an excellent prognostic molecular signature of peace was developed. The ferroptosis-based molecular signatures ASNS, GPT2, NFE2L2, and RRM2 were combined to develop a powerful prognostic molecular signature for PCa.

## Conclusions

We implemented a new ferroptosis-based signature for PCa RFS using four FRGs identified by univariate and multivariate Cox regression analyses. The signature was validated in training, test and validation cohorts, with excellent performance based on ROC curves of 0.757, 0.715 and 0.732, respectively. Furthermore, we found that younger patients or those with stage T III and T IV, stage N0, cluster 1 and cluster 2 were best predicted by the signature as independent predictors of RFS. DU-145 and RWPE-1 cell lines were successfully analysed by qRT-PCR and Western blot for ASNS, GPT2, RRM2 and NFE2L2.

## Materials and methods

### Data collection

We downloaded transcriptome profiles of 497 PCa cases and 52 normal cases from the Cancer Genome Atlas (TCGA) database. For the 70 cases data on "Recurrence_days_between_biochemical_recurrences", we used it to be the time to cancer recurrence (CR). For those without this data type, we used "A8_New_Event_Time" as the recurrence time. We preferred to use "Type_A8_New_Event" as the recurrence-free survival (RFS) status. If "Type_A8_New_Event" data was not available, we defined "biochemical recurrence" as the RFS. From extreme care to the initial RFS or demise, the RFS was delineated. TCGA database has complete transcriptome information, RFS status, and RFS time for 405 PCa cases. GSE70768, containing 199 PCa cases with full transcriptome and RFS data, was procured from the GEO database. Additionally, we downloaded 382 FRGs from the FerrDb database, including 150 ferroptosis activators, 109 ferroptosis suppressors, and 123 ferroptosis markers.

### Differentially expressed FRGs and functional enrichment

The "Lima" R package was employed to analyze a TCGA cohort of 382 FRGs, and the Wilcoxon test was then employed to distinguish DEFRGs in TCGA samples that diverged between normal and PCa samples, with an FDR (false discovery rate) cutoff of < 0.05 and a log2|fold change (FC) |> 0.5. A heatmap was then generated with the "pheatmap" R package to illustrate all DEFRGs in PCa. Utilizing the R packages "cluster Profiler" and "org. Hs. Aug. deb", functional enrichment of Gene Ontology (GO) and Kyoto Encyclopedia of Genes and Genomes (KEGG) were achieved.

### Molecular clusters of ferroptosis identified by consensus clustering

We proceeded to perform univariable Cox regression analysis to identify DEFRGs associated with RFS as potential prognostic factors. A p-value of less than 0.05 was established as the significance level. Subsequently, we employed consensus clustering analysis using the R package "ConsensusClusterPlus" two divided patients into different molecular subtypes of ferroptosis related. We performed the R packages "survival", "survminer", as well as "pheatmap" to explored the association between these molecular markers and Clinico-pathological character, such as RFS, stage T and stage N. The PD-L1 cx gene expression level was also investigated in relation to ferroptosis-related molecular clusters.

### RFS signature development and verification using ferroptosis

We randomly divided the TCGA cohort into two groups, one for training and one for testing, with 405 and 201 cases respectively, to create a ferroptosis-based marker for forecasting RFS. Both groups had comparable clinicopathological data. The GEO cohort of 199 PCa cases was also used as a validation cohort. We conducted univariate and multivariate Cox regression analyses on the training cohort, with a cutoff p value of 0.05, dividing PCa patients into high-risk and low-risk groups based on the median risk score from the signature. Subsequently, we validated the signature's performance through survival analysis, receiver operating characteristic (ROC) curve analysis, and independent prognostic analysis. Furthermore, we validated the signature's performance in both the testing and validating cohorts.We employed the "pheatmap" R package to present the risk distributions for the TCGA cohort, training cohort, testing cohort, and validating cohort, as well as to conduct ROC curve analysis and survival analyses. Additionally, we conducted subgroup survival analyses in stage T I and T II, stage T III and TIV patients, and stage N0 and N1 PCa patients, with a p value of less than 0.05 deemed statistically significant.

### A correlation between ferroptosis-based signatures and functional enrichment

We delved deeper into the connection between the novel ferroptosis-based marker and its underlying mechanisms in this study. To further explore this, we employed gene set enrichment analysis (GSEA) as previously mentioned^20^.

### qRT-PCR

qRT-PCR was conducted on DU-145(prostate cancer cell line) and RWPE-1(normal prostatic epithelial cell line) cells to validate the different mRNA expression levels of risk DEFRGs. The TRIzol reagent (Invitrogen, Carlsbad, CA) was employed to extract the total RNA of the risk DEFRGs, while a NanoDrop 2000 spectrometer (Thermo Fisher Scientific, Waltham, MA) was utilized to ascertain RNA purity. TransScript^®^ Green One-Step QRT-PCR SuperMix (TransGen Biotech, Beijing, China) was then employed to execute reverse transcription reactions in a single step. Using the Qaq Pro universal SYBR qPCR Master Mix (Vazyme, Nanjing, China), the four gene expression levels were determined, as per the manufacturer's protocol. Actin expression was then normalized to the results.

### Western blot

Validation of the varying protein expression levels of DEFRGs was accomplished through the utilization of RWPE-1 and DU-145. As per the manufacturer's protocol, (1) Sample preparation: Protein samples were removed, 4 × SDS loading buffer was added to a final concentration of 1×, water was added to a total volume of 15 μl and denatured in boiling water at 100 °C for 5 min. (2) Tricine-SDS-PAGE electrophoresis gel: prepare separation gel (containing 10% glycerol) and stacking gel. (3) Electrophoresis: Electrophoresis after sample addition was performed at a constant voltage of 60 V for the stacking gel and 140 V for the separation gel, and 30 min (4) Membrane transfer: NC membrane was used to transfer the membrane at a constant current of 200 mA for 1.5 h. (5) To transfer the membrane, a constant current of 200 mA was employed for 1.5 h, and (6) 5% BSA was used to block the NC membranes for 1 h at room temperature. Lastly, the primary antibody was added overnight at 4 °C (7) Membrane washing: TBST was washed three times; TBS was washed once for 5 min (8) After three successive washings of TBST and a single wash of TBS for 5 min, secondary antibody was incubated at room temperature for 1 h in the dark. (9) Membrane washing: TBST was washed three times; TBS was washed once for 5 min (10) Development: visualization was performed using an Odyssey CLx Imager detection instrument and analyzed by Image Studio software.

### Supplementary Information

Supplementary Information.

## Data Availability

The datasets generated and/or analysed during the current study are available in the [TCGA] repository, [https://portal.gdc.cancer.gov/] and [GEO] repository, [https://www.ncbi.nlm.nih.gov/geo/query/acc.cgi?acc=GSE70768].
