# Supplementary material for: Development of a ferroptosis-based molecular markers for predicting RFS in prostate cancer patients
Source: Sci Rep. 2023 Dec 20;13:22804. doi: 10.1038/s41598-023-50205-1 (PMC10739732; doi:10.1038/s41598-023-50205-1)
Supplement: Supplementary file 1 — Supplementary Information. [file 41598_2023_50205_MOESM1_ESM.pdf]

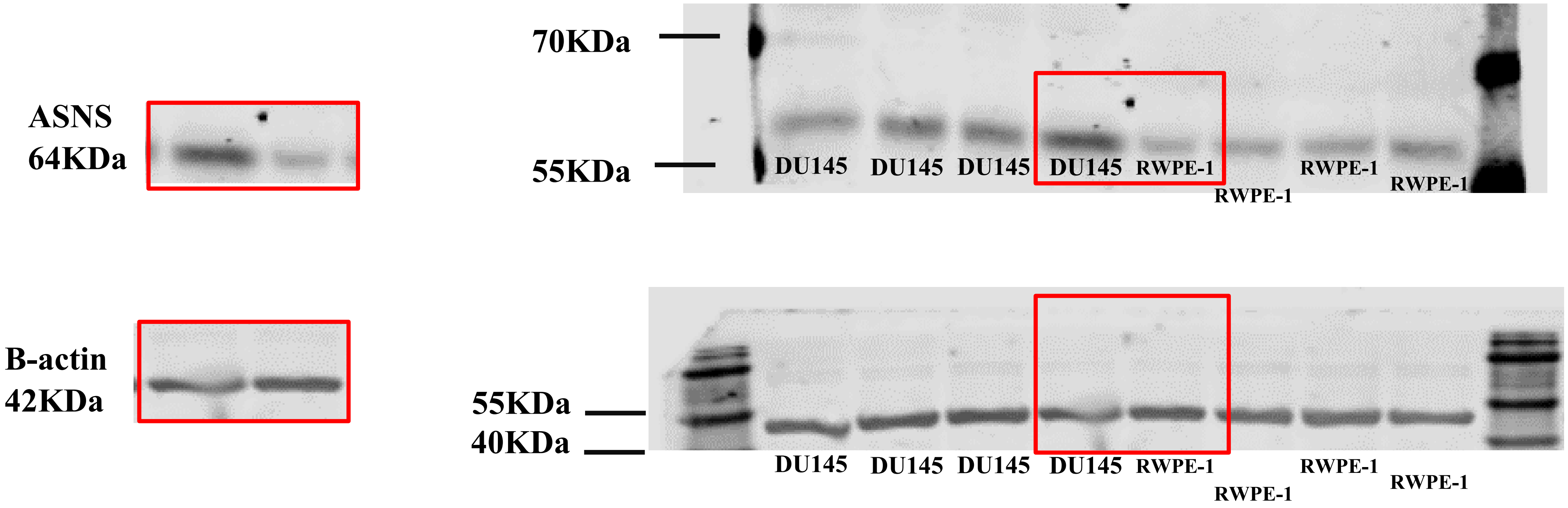

Supplementary Gel for Figure 8A (upper panel in DU145 and RWPE-1 cells)

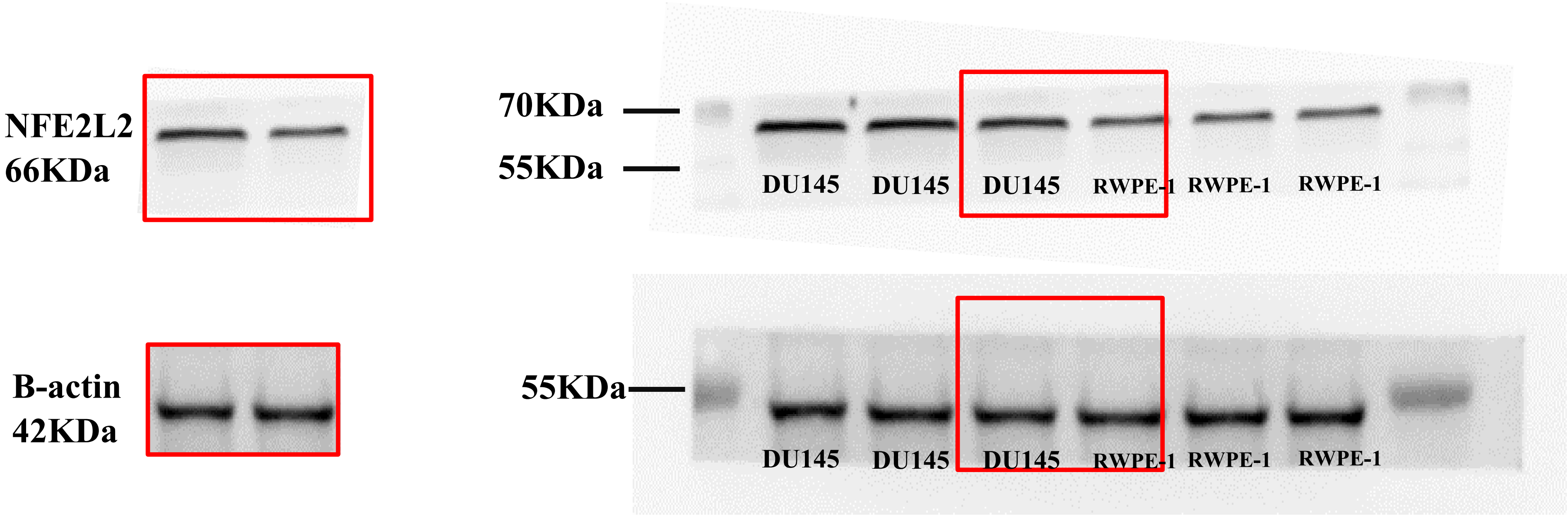

Supplementary Gel for Figure 8B (upper panel in DU145 and RWPE-1 cells)

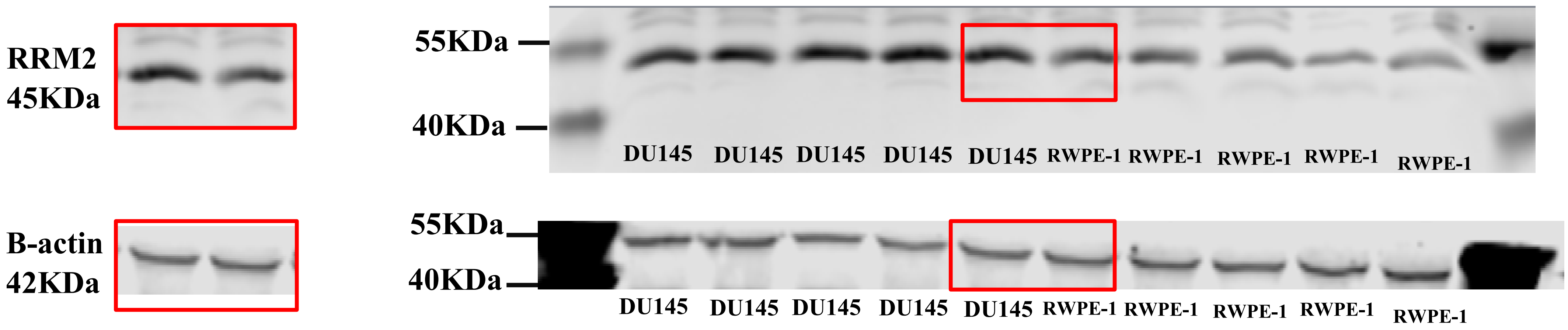

Supplementary Gel for Figure 8C (upper panel in DU145 and RWPE-1 cells)

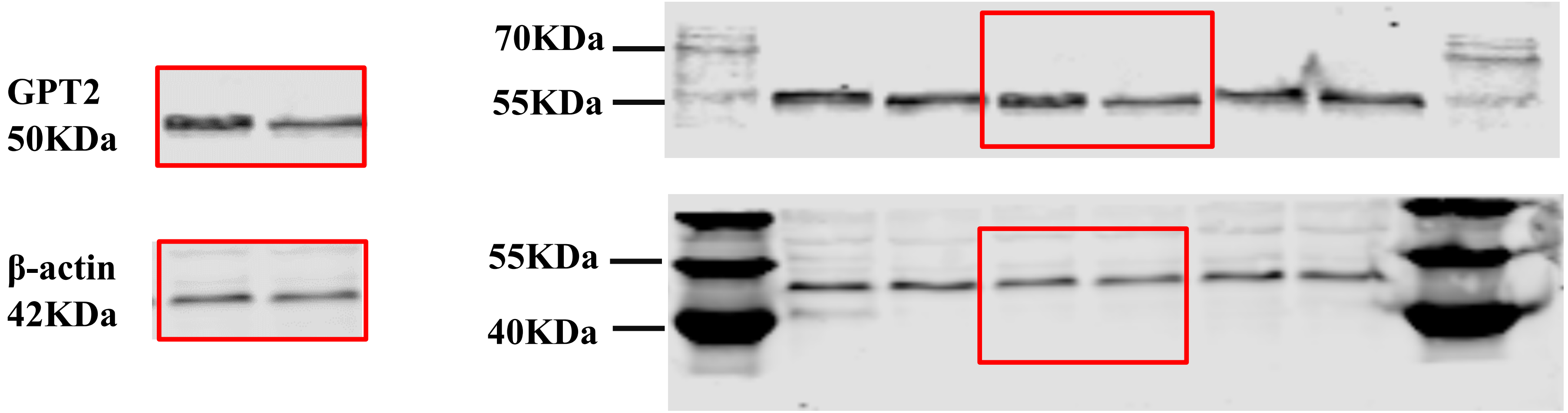

Supplementary Gel for Figure 8D (upper panel in DU145 and RWPE-1 cells)
